# An Interdisciplinary Ecosystem for the Prevention of Cardiotoxicity in Older Patients With Breast Cancer: Protocol for a Prospective and Multicentric Study

**DOI:** 10.2196/63455

**Published:** 2025-08-14

**Authors:** Gaia Giulia Angela Sacco, Ketti Mazzocco, Anastasia Constantinidou, Andri Papakonstantinou, Davide Mauri, Grigorios Kalliatakis, Manolis Tsiknakis, Domen Ribnikar, Dorothea Tsekoura, Valantis Aidarinis, Kalliopi Keramida, Panagiotis Oikonomopoulos, Athos Antoniades, Cameron Brown, Federica Rizzi, Anca Bucur, Elsa Pacella, Georgia Karanasiou, Daniela Cardinale, Carlo Cipolla, Elisabetta Munzone, Dimitrios Fotiadis, Giuseppe Curigliano, Gabriella Pravettoni

**Affiliations:** 1 European Institute of Oncology, IRCCS Milan Italy; 2 Department of Oncology and Hemato-oncology, University of Milan Milan Italy; 3 Medical Oncology Department, Bank of Cyprus Oncology Centre Nicosia Cyprus; 4 Oncology-Pathology Dept., Karolinska Institutet - Cancer Center Karolinska (CCK) Solna Sweden; 5 Medical Oncology Department, General Hospital of Lamia Lamia Greece; 6 Computation, Computational BioMedicine Laboratory (CBML), Foundation for Research and Technology-Hellas (FORTH) Heraklion Greece; 7 Department of Electrical & Computer Engineering, Hellenic Mediterranean University (HMU) Chania Greece; 8 Medical Oncology Department, Institute of Oncology Ljubljana Ljubljana Slovenia; 9 2nd Department of Surgery, Aretaieio University Hospital, National and Kapodistrian University of Athens Athens Greece; 10 2nd Department of Cardiology, Attikon University Hospital, National and Kapodistrian University of Athens Athens Greece; 11 Stremble Ventures LTD Limassol Cyprus; 12 IMS - Istituto di Management Sanitario Bresso Italy; 13 Philips Research Europe Eindhoven The Netherlands; 14 ESC, European Society of Cardiology Sophia Antipolis France; 15 Department of Materials Science and Engineering, UOI - University of Ioannina Ioannina Greece

**Keywords:** breast cancer, older adult, cardiotoxicity, prospective study, eHealth, mobile app, risk prediction model

## Abstract

**Background:**

Over 50% of newly diagnosed patients with breast cancer are aged ≥65 years. Due to age-related factors and the presence of comorbidities, these patients are particularly vulnerable to developing cardiac toxicity associated with cancer treatments, which may lead to suboptimal interventions and undertreatment, resulting in poorer health outcomes, quality of life (QoL) deterioration, and increased health care costs. Given the underrepresentation of older patients with breast cancer in clinical trials and the increasing recognition of the impact of psychosocial and behavioral factors on cardiovascular disease onset, broader and interdisciplinary studies are required to develop new and innovative best practices for this clinical population.

**Objective:**

Using an innovative eHealth approach combining the CARDIOCARE (An Interdisciplinary Approach for the Management of the Elderly Multimorbid Patient with Breast Cancer Therapy Induced Cardiac Toxicity—grant agreement 945175) mobile app and technologically advanced wearable devices (ie, the Garmin Venu SQ watch and Polar H10 sensor), the CARDIOCARE prospective study pursues a twofold aim: (1) testing the effectiveness of the CARDIOCARE mobile app to monitor and assess the intrinsic capacity and QoL of older patients with breast cancer and evaluating the CARDIOCARE eHealth interventions’ effectiveness on these parameters and (2) developing a holistic, patient-centered risk prediction model specific for the detection of cardiotoxicity before it clinically emerges.

**Methods:**

This prospective and multicentric study involves 6 clinical and 5 technical partners across Europe. In total, 750 older patients with breast cancer (aged ≥60 years) are assigned to either the standard practice or enhanced monitoring group, with only patients in the latter receiving access to eHealth psychological, behavioral, and functional interventions implemented on the CARDIOCARE eHealtHeart app. Patients will be recruited in 6 clinical centers and will undergo clinical procedures to collect multimodal data, including clinical data, cardiac imaging, biochemical and psychological biomarkers and omics, intrinsic capacity, and QoL indicators measured at baseline (T0) and every 3 months up to 12 months (T5).

**Results:**

CARDIOCARE is a project funded by Horizon 2020, and enrollment started in May 2023. As of October 17, 2024, a total of 50% (375/750) of the target number of patients had been recruited.

**Conclusions:**

The CARDIOCARE prospective study will contribute to developing new best practice guidelines for managing older patients with breast cancer and multimorbidity while preserving their intrinsic capacity and improving their QoL. Furthermore, the CARDIOCARE mobile app and the wearable devices will allow clinicians to identify trajectories across the cardiotoxicity disease continuum and thus intervene in a preventative way among patients at higher risk. Such a health care approach will also benefit the health care system, which currently spends almost 40% of its resources on patients aged >60 years, with long-term care and hospital admissions being the primary cost drivers.

**Trial Registration:**

ClinicalTrials.gov NCT06334445; https://clinicaltrials.gov/study/NCT06334445

**International Registered Report Identifier (IRRID):**

DERR1-10.2196/63455

## Introduction

### Background

Over 50% of newly diagnosed patients with breast cancer are aged >65 years and are particularly vulnerable to the cardiotoxic effects of cancer treatment due to multiple comorbidities and age-related risk factors [1]. The cumulative effect of risk factors in the older patient with breast cancer resembles a “snowball effect” in which baseline age-related risk factors and cancer-related changes are further exacerbated by cancer therapy–induced cardiotoxicity [2,3], resulting in severe multimorbid conditions and increased mortality. Underestimating the cardiotoxicity risk in this vulnerable population may lead to inappropriate interventions and undertreatment, contributing to poorer health outcomes, quality of life (QoL) deterioration, and increased health care costs. Considering that older patients with cancer are systematically underrepresented in clinical trials [1], there are currently limited means to effectively address the complex needs of these patients and their caregivers, often resulting in undertreatment and suboptimal health outcomes, with negative consequences to patients’ QoL [1]. Therefore, in line with the World Health Organization’s view that health is a state of complete physical, mental, and social well-being [4], broader, interdisciplinary, and patient-oriented clinical trials are urgently needed to provide new evidence-based best practices for managing older patients with breast cancer. To date, the most effective approach to minimize cardiotoxicity is baseline risk stratification and appropriate follow-up during treatment for the early detection of cardiotoxicity.

Moreover, psychological conditions such as depression, distress, and anxiety—often observed in patients with cancer after diagnosis [5]—can increase the risk of cardiovascular complications [6]. Similarly, people presenting negative affectivity and social inhibition are at higher risk of cardiovascular disease as such negative affectivity is significantly associated with higher systolic and diastolic blood pressure [7,8]. Similarly, prolonged exposure to stressful life circumstances may predict subsequent hypertension and cardiovascular disease [3]. Therefore, the Integrated Care for Older People guidelines recommend a comprehensive cardiological assessment as well as a detailed evaluation of patients’ intrinsic capacity. Intrinsic capacity refers to the composite of all the physical and mental capacities that an individual can draw upon at any point in time. It encompasses a wide range of abilities, including cognitive, emotional, physical, and sensory functions, that together influence an individual’s functional ability and QoL as they age [9]. This holistic and patient-oriented approach aims to establish new best practices for the management of older patients with cancer at risk of cardiotoxic effects associated with oncological treatments [10].

### Novelty and Study Aim

Multimodal retrospective data have been retrieved from existing databases in 5 of the 6 clinical centers belonging to the CARDIOCARE consortium and involved in the observational prospective cohort study (hereafter referred to as the *prospective study*)—the Bank of Cyprus Oncology Centre (BOCOC) in Cyprus, the European Institute of Oncology (IEO) in Italy, Karolinska University Hospital (KSBC) in Sweden, National and Kapodistrian University of Athens (NKUA) in Greece, and University of Ioannina (UOI) in Greece—to develop a risk prediction model for cardiotoxicity in the older adult population with breast cancer to be refined and validated through the CARDIOCARE prospective study. In the prospective study, the cardiotoxicity risk prediction model will be enriched with novel biochemical, omics (metagenomics, micro-RNAs, and single-nucleotide polymorphisms), and psychological markers to better assess how patients’ intrinsic capacity (physical and mental) and QoL are affected by cancer and anticancer treatments and how they influence the risk of developing cardiac toxicity. So far, no extensive studies have allowed for the collection of all the necessary data to conduct a meaningful assessment of the intrinsic capacity and QoL of older patients with breast cancer.

It has to be considered that patients recruited in the CARDIOCARE clinical study receive different types of systemic therapy, including chemotherapy, targeted therapy, immunotherapy, and hormonal therapy. They also receive different types of local treatment comprising surgical treatment (wide local excision vs mastectomy) as well as radiotherapy. The aim is indeed to explore the impact of each treatment separately (different categories) and present potential associations between therapies and QoL and intrinsic capacity parameters. In standard clinical practice, these are managed through a “one-size-fits-all” approach, but CARDIOCARE, focusing on personalization of care, may ultimately change that.

Indeed, different pharmacological interventions can cause different types of cardiotoxicity and side effects, hence varying the impact on QoL. Data on the individual impact of the different types of systemic cancer therapy on intrinsic capacity are scarce, and the CARDIOCARE platform provides a unique opportunity to explore this as aggregated data and separately.

Thus, the CARDIOCARE prospective study will adopt technologically advanced tools to collect a large amount of data on the physical, psychosocial, and behavioral parameters of older patients with breast cancer to better identify patients at risk of developing cardiotoxicity due to oncological treatments and improve their psychological and physical well-being during and after their oncological care pathway.

Monitoring technologies based on sensors, wearables, eHealth (ePsycHeart and eHealtHeart), and a mobile app are used to collect patient-reported data; engage patients in their ongoing health and behavior; and make an impact on the communications between patients and clinicians in a closer, more trusting environment.

The mobile app is structured in 2 different main sections: the ePsycHeart app and the eHealtHeart app. This app is used during the first 6 months of the study to collect psychological, social, and physical variables.

The ePsycHeart section has been developed with a patient-oriented approach aiming to easily monitor and inform patients and physicians on the patients’ overall emotional, functional, and physical well-being by means of smart recommendations and on the most adequate intervention activity (eHealtHeart), tailored based on the output originated from ePsycHeart. In particular, ePsycHeart will contain different validated and standardized questionnaires to assess psychological, social, and physical variables.

The eHealtHeart section includes the main interventions, developed in modules to counteract dispositional, emotional, and behavioral risk factors for cardiotoxic effects and lower QoL after cancer therapy. Specifically, eHealtHeart will deliver effective interventions to prevent and delay progression of cardiotoxicity by targeting (1) the improvement of psychocognitive well-being, (2) the improvement of mobility and vitality, (3) the management of age-associated conditions (eg, urinary incontinence), (4) the prevention of falls, and (5) support for caregivers. The eHealtHeart main modules focus on several aspects.

One of the modules targets psychological well-being, with interventions on depression, emotional and dispositional states, and stressors based on a number of intervention tools. The first tool is biofeedback, which is a well-established method for collecting the user’s physiological parameters to foster better outcomes in health care, understanding the disease and delivering treatment. More specifically, through noninvasive external sensors, biofeedback gives constant real-time feedback to the user about parameters such as heart rate (HR). This feedback allows the patient to gain awareness and learn how to self-regulate their own bodily reactions, which are usually unconscious and automatic, through individualized approaches that improve one’s adaptability to physiological responses [11,12].

The second tool is the best possible self (BPS) activity, which was introduced by King [13] as a disclosure exercise in which participants write about their life goals and priorities imagining themselves in the future once everything has turned out for the best. Participants will be asked to carry out this task for 4 or 5 days every month for the first 6 months of the study. BPS is a writing paradigm developed to help participants set goals and priorities for a positive, imagined future [14], with strong evidence of improving mental and physical well-being.

The third tool is expressive writing, a cost-effective therapeutic intervention in which individuals are asked to write for approximately 15 to 20 minutes about a major life event or traumatic experience over 3 or 4 consecutive days. Research has reported reductions in biological indicators of stress and stress-related conditions affecting blood pressure, sleep, depression, immune function, and pain [15].

The fourth tool is ABCDE, a method in which each participant is asked to think about an adversity (A) that happened during the last period. The participant is then asked to return to the thoughts they had while facing the adversity and reflect on their own beliefs associated with that adversity (B). Successively, the participant is invited to focus on the consequences (C) of their beliefs in terms of experimented feelings and actions taken. After that, the participant needs to dispute (D) the beliefs, meaning that the person’s beliefs will be attacked and questioned. Finally, the last step is called Energy I, and it helps the participant observe the connection between the thought (B) and the consequences (C). This last step should help the participant gain awareness about the fact that pessimistic thoughts lead to negative reactions and emotions, whereas positive beliefs lead to positive outcomes regarding one’s own feelings as well as environmental consequences.

Another eHealtHeart module focuses on cognitive stimulation and training via find the word, tic-tac-toe, color beans, and drawing exercises. Every 6 months, the cognitive effect of these exercises on patients will be assessed.

Improvement of mobility and vitality are addressed by another eHealtHeart module. The physical activity and exercise module offers a collection of different graphs, such as bar, line, and pie charts, that will represent data collected through a physical activity–tracking device. The monitoring of steps, duration of sleep, and daily summaries (exercise, type of activity, average HR, and stress level) will be presented to inform patients and physicians about the overall functional well-being of the patient.

Sensory screening is also conducted in the eHealtHeart main modules. A vision test that follows the Snellen test guidelines will be available. The patient must keep the mobile phone screen at a certain distance and, by closing each eye, must assess the letters that the mobile app shows. At the end, an overall score is provided based on the patient’s answers. The Snellen test can be repeated as many times as the patient wants within a day. A hearing test that follows the whisper test guidelines will also be available. The procedure will play back certain sounds from the left to the right side of the smartphone speakers. On the basis of what the patient heard and did not hear, an overall score with scaling is provided. The whisper test can be repeated as many times as the patient wants within a day.

The eHealtHeart main modules also promote the management of geriatric syndromes. In the Incontinence Management module, a patient will be able to report urination incidents, follow instructions to exercise the pelvic muscles, and add reminders regarding pelvic floor exercises. The urination data will be stored on the CARDIOCARE server and will be available to the patient and the patient’s assigned health care professional. Furthermore, the patient will be prompted to exercise their pelvic floor muscles with the help of instructions and visual aids. The pelvic floor exercises have different difficulty levels (regarding, eg, repetitions and time).

A fall detection and fall prevention module will be available on the app. The system, using sensors from the mobile device, will try to detect whether the patient had a fall incident and will inform the caregiver to communicate with the patient. Furthermore, educational material containing guidelines and videos for fall prevention will be available to the patients.

On the first day of recruitment, each patient receives a mobile phone (with the CARDIOCARE app), a smartwatch, and a belt from their clinical center to be used during the first 6 months of the study. Details on the frequency and duration of the interventions are provided in Table 1.

**Table 1:** Frequency of the interventions of the eHealtHeart mobile app.

Module and interventions		Frequency
**Psychological well-being measured until month 6**		
	Biofeedback	If the patient reports a high anxiety and depression value in the ePsycHeart section (PHQ-4^a^), an alert will suggest to complete the module.
	BPS^b^	20 minutes each day for 4-5 consecutive days every 3 months starting 1 month after baseline
	EW^c^	20 minutes each day for 4-5 consecutive days every 3 months starting at baseline
	ABCDE^d^	Every 3 months starting 2 months after baseline
**Cognitive stimulation and training**		
	Find the word Tic-tac-toe Color beans Drawing	5 consecutive days per month until month 6
**Improvement of mobility and vitality**		
	Physical activity and exercise	At least 48 continuous hours each week until month 6
**Sensory screening**		
	Vision test Hearing test	At baseline and at month 6
**Management of geriatric syndromes**		
	Pelvic floor exercises	Daily for 3 months
	Fall detection and fall prevention	Twice a week for 6 weeks

^a^PHQ-4: 4-item Patient Health Questionnaire.

^b^BPS: best possible self.

^c^EW: expressive writing.

^d^ABCDE: activating event or situation, beliefs, consequences, disputation of beliefs, and effective new approach.

### Objectives and Rationale

The objectives and rationale of this prospective clinical study are as follows:

1. To stratify patients aged ≥60 years with breast cancer based on their risk of developing cardiotoxicity. The rationale is that, so far, multiple studies have explored the effects of specific risk factors; however, these studies have yet to deal comprehensively with the impact of clinical, biological, psychological, and behavioral risk factors and their interactions.
2. To evaluate the effects of eHealth psychological and behavioral interventions on the intrinsic capacity and QoL of older patients with breast cancer and on their risk of developing cardiac toxicity. The rationale is that, although recent studies associate psychosocial and behavioral characteristics with the risk of developing cardiovascular diseases, these studies on older patients with breast cancer are still limited [16]. However, gaining such knowledge is necessary to generate hypotheses on using eHealth interventions to support this clinical population.

The primary study end point is to evaluate and monitor the onset of cardiotoxicity, where subclinical cardiotoxicity is defined as preserved left ventricular ejection fraction (LVEF; ie, LVEF≥50%) with >15% reduction in global longitudinal strain relative to the baseline or new rise in cardiac troponin I or natriuretic peptide levels in plasma and clinical cardiotoxicity is defined as the reduction in LVEF by ≥10% from baseline to a value of <50% at any time point during study follow-up [17,18].

Secondary end points include intrapatient assessment of major adverse cardiovascular events (MACEs) [19,20] (angina pectoris, acute myocardial infarction, major ventricular arrhythmias, pacemaker implantation, acute heart failure [New York Heart Association class 4], acute ischemic brain disease with neurological persistence evidence, and severe hypertension requiring treatment with angiotensin-converting enzyme or β-blockers), intrapatient assessment of plasma troponin I levels during the follow-up and their association or agreement with LVEF, intrapatient assessment of plasma brain natriuretic peptide elevation, hospital admissions due to cardiovascular causes or falls, cardiovascular death, noncardiovascular death, and health-related QoL (assessed using the European Organisation for Research and Treatment of Cancer Quality of Life Questionnaire–Breast Cancer, a validated breast cancer patient-reported outcome measure).

Secondary end points also include the cost-effectiveness of provided health care pathways, determined through costs combined with quality-adjusted life years (QALYs). Costs will consider health care provided, numbers of admissions and days spent in hospital, and patient costs for out-of-pocket expenses associated with their condition (ie, travel expenses [of both the patient and caregiver], over-the-counter medicines and supplements, complementary therapies not supported by the health care system, home help, and time away from work).

Psychological and behavioral variables will also be measured as secondary end points. Intrinsic (mental and physical) capacity will be evaluated through the comprehensive geriatric assessment using standardized patient-reported outcome measures where applicable, sensors and wearables, and performance tests. More specifically, the following variables will be measured—(1) mobility and locomotion (distance, balance, and gait speed), (2) sensory ability (Snellen test and whisper test), and (3) vitality status (exercise, electrocardiogram, HR variability, grip strength, nutritional or energy state, sleep, and fatigue)—together with a complementary frailty screening (using the Geriatric 8 frailty screening tool, declared as the preferred tool by the International Society of Geriatric Oncology for identifying frailty in older patients with cancer, including patients with breast cancer) and frailty assessment (comorbidity, mental health, cognition, functional status, polypharmacy, geriatric syndromes, and socioeconomic and nutritional status). Other psychological and behavioral variables measured are psychocognitive status (personal traits, cognitive factors, and emotional states), social and socioeconomic factors, and general QoL, measured using standardized validated questionnaires to make comparisons with the more specific psychological and behavioral measures listed previously.

The following continuous variables will also be measured to identify predictive factors of disease trajectories and cardiac toxicity related to the objective of developing, refining, and validating the risk stratification model. Imaging markers of cardiac structural and functional variables will be measured using echocardiography, including LVEF percentage (Simpson biplane method [left ventricle end-diastolic volume and left ventricle end-systolic volume]), left ventricular diastolic dysfunction+left ventricular systolic dysfunction, interventricular septal end diastole, left ventricular posterior wall end diastole, fractional shortening percentage, mass index+relative wall thickness, tricuspid annular plane systolic excursion+S′ tricuspid annulus, left ventricular outflow tract velocity time integral+left ventricular outflow tract maximum velocity+aortic velocity time integral+aortic maximum velocity+aortic dimension, early to late diastolic transmitral flow velocity+medial early diastolic mitral annulus velocity +systolic-to-diastolic duration ratio, maximal tricuspid regurgitation velocity, right atrial pressure, left atrial volume index+right atrial volume index, global longitudinal strain (of both ventricles if possible), and myocardial work (global work index, global constructive work, global wasted work, and global work efficiency if available)+systolic and diastolic pressure. In addition, 2D echocardiographic sequences (grayscale images or videos of at least one representative full cardiac cycle) will be extracted as Digital Imaging and Communications in Medicine (DICOM) files for 3 different views (4 chambers, 3 chambers, and 2 chambers); independent 2D grayscale static images at end-diastole and end-systole frames will be extracted as DICOM files for each view; and corresponding manual annotations of the left ventricle (or left endocardium), left epicardium, and left atrium will be extracted from a subset of the aforementioned provided images.

Mammography DICOM images corresponding to the left mediolateral oblique view, right mediolateral oblique view, left craniocaudal view, and right craniocaudal view (conventional or tomosynthesis) will be obtained if the patient did not undergo mastectomy.

Other continuous variables that will be measured are whole-blood and plasma biomarkers, including biochemical (troponin I and brain natriuretic peptide), inflammatory and psychological (eg, if available, interleukin-6, tumor necrosis factor, HR variability, C-reactive protein, fibrinogen, and ferritin), and omics (single-nucleotide polymorphisms and extracellular vesicle micro-RNAs) biomarkers, and microbiome biomarkers from metagenomic analysis of stool samples (species diversity indexes; relative abundance at the family, genus, and species levels; and presence of pathogenic species).

## Methods

### Partners

The CARDIOCARE prospective study is multicentric and involves 6 European clinical partners: the IEO—the study clinical coordinator—in Italy, the BOCOC in Cyprus, the KSBC in Sweden, the UOI and NKUA in Greece, and the Institute of Oncology Ljubljana (IOL) in Slovenia. The work of these clinical centers, and especially the technological aspects of eHealth tool implementation, is supported by 5 European technical partners: the Foundation for Research and Technology, the Hellenic Mediterranean University, and the UOI, all based in Greece; Stremble, a research and development company based in Cyprus offering a range of advanced analytics, bioinformatics, and software engineering expertise; the European Society of Cardiology based in France; and the Philips Electronics Nederland B.V. company in the Netherlands. The Institute of Health Management in Italy is also part of the CARDIOCARE consortium, ensuring the highest level of study conduct and management quality.

### Study Design

This multicenter clinical study has been designed as a prospective observational study that examines whether integrated patient-oriented psychological, behavioral, and functional interventions can prevent, delay, or mitigate cardiotoxicity as an adverse effect of cancer therapy. It will include 750 older patients with breast cancer recruited across the participating clinical centers.

All recruited patients will be randomly assigned to either the standard practice or enhanced monitoring group. Patients in both arms, in addition to standard oncological care, will receive eHealth monitoring through the Garmin Venu SQ and Polar H10 technologically advanced wearable devices (described in detail in the Instruments and Measures section) and will have access to the ePsycHeart assessment tool on the CARDIOCARE mobile app (described in detail in the Instruments and Measures section). Furthermore, patients will be given a Motorola smartphone on which the CARDIOCARE mobile app is installed. These technological instruments will provide clinicians with the patients’ physical and psychological intrinsic capacity and QoL indicators as foreseen in the study protocol. However, only patients in the intervention arm will have access to the behavioral, psychological, functional, and educational interventions in the eHealtHeart section of the CARDIOCARE mobile app, which will allow researchers to evaluate the effectiveness of such eHealth interventions on the intrinsic capacity and QoL of older patients with breast cancer, as well as these interventions’ effectiveness in mitigating, delaying, or even preventing the onset of cardiac toxicity associated with breast cancer treatments.

As per the protocol, before undertaking any study procedure, the patients will receive a patient information sheet, in which the different study procedures are explained in lay language. The same document also sets out the study’s risks, benefits, and aims. Once the patient has read the patient information sheet and has received answers to any questions or concerns, she can choose to provide informed consent to participate in the study by signing the written informed consent form, of which she will be left with a copy, whereas the CARDIOCARE researchers will keep the original document.

**Figure 1:**
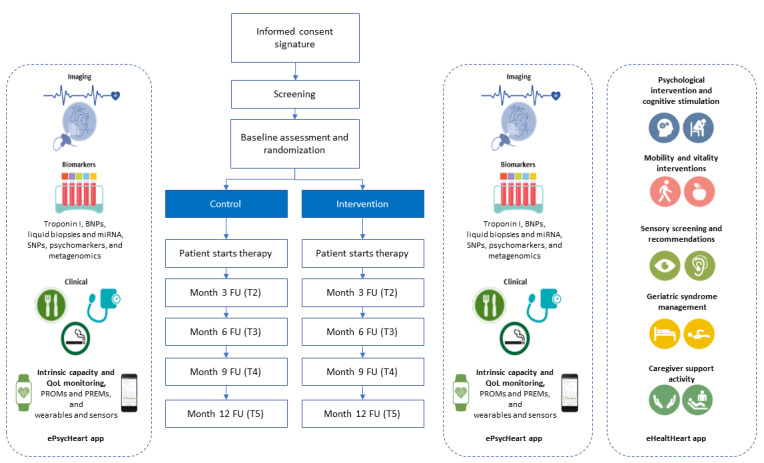
Study design. BNP: brain natriuretic peptide; FU: follow-up; PREM: patient-reported experience measure; PROM: patient-reported outcome measure; QoL: quality of life; SNP: single-nucleotide polymorphism.

Patients will be assigned through the study electronic case report form (eCRF) at baseline to the standard practice or enhanced monitoring group in a 1:1 ratio. The study time points, follow-ups, and specific procedures are detailed in the Study Time Points and Procedures section (Figure 1).

Great attention has been paid to the standardization of procedures among the clinical centers involved. Specifically, the inclusion criteria for the study are thoroughly detailed and are consistent across all 6 clinical centers involved in recruitment. This ensures that the same standards are followed at each site, allowing for a homogeneous participant selection process.

In addition, the technical partners involved in the study are responsible for continuously monitoring the data entered into the eCRF. They actively identify any values that may require further scrutiny or validation, ensuring the quality and integrity of the data.

Furthermore, the CARDIOCARE platform plays a crucial role in the standardization process. It provides real-time monitoring of whether patients are adhering to the prescribed activities according to the study protocol. If any discrepancies are detected, the clinical centers can contact their patients to address any issues, ensuring that all sites are aligned with the study protocol.

Therefore, while working across multiple centers presents certain challenges, the combination of detailed inclusion criteria, constant monitoring through eCRFs, and checks via the CARDIOCARE platform makes the process both feasible and practical. This multilayered approach allows for high levels of standardization and consistency across all sites.

Potential sources of bias have been minimized as follows. Regarding recall bias, the same questions are asked in both groups, ensuring that the bias is homogeneous, and reminders are sent to patients to complete the questionnaire on time. Regarding selection bias, the inclusion criteria are stringent, and all suitable candidates are enrolled by the centers. Regarding confirmation bias, the primary end points and most secondary end points are quantitative, reducing the likelihood of interpretation errors. Regarding observation bias, some questions are repeated across questionnaires, and patients complete the same questionnaire 3 to 5 times during the study.

### Study Participants

A total of 750 women aged ≥60 years with a diagnosis of breast cancer will be recruited across the 6 European clinical centers involved in the CARDIOCARE consortium in Italy, Greece, Cyprus, Sweden, and Slovenia (IEO: n=125, 16.7%; BOCOC: n=120, 16%; KSBC: n=125, 16.7%; UOI: n=60, 8%; NKUA: n=195, 26%; IOL: n=125, 16.7%). A subsample of 500 patients will be randomly selected (IEO: n=100, 20%; BOCOC: n=100, 20%; KSBC: n=80, 16%; NKUA: n=130, 26%; IOL: n=90, 18%) to allow Stremble to assess gut microbiome changes associated with oncological treatments. Details on the inclusion and exclusion criteria to recruit patients are provided in the Selection Criteria section.

### Selection Criteria

#### Inclusion Criteria

The inclusion criteria are as follows: (1) women aged ≥60 years with a diagnosis of early or locoregional breast cancer who will undergo neoadjuvant or adjuvant treatment with regimens including anthracyclines or taxanes; (2) women aged ≥60 years with a diagnosis of human epidermal growth factor receptor 2 (HER2)–positive early or locoregional breast cancer who will undergo neoadjuvant or adjuvant treatment with anti-HER2 therapy (trastuzumab or trastuzumab and pertuzumab); (3) women aged ≥60 years with a diagnosis of early or locoregional breast cancer who will undergo neoadjuvant or adjuvant treatment with endocrine therapies with or without cyclin-dependent kinase 4 or 6 inhibitors; (4) women aged ≥60 years with HER2-positive metastatic breast cancer who will undergo first-line anti-HER2 therapy (trastuzumab or trastuzumab and pertuzumab with or without chemotherapy); (5) women aged ≥60 years before starting the aforementioned treatment for breast cancer; (6) eligible women aged ≥60 years who will undergo first-line therapy in the metastatic setting with any type of treatment (chemotherapy, immunotherapy, or biological agents); (7) willingness and ability to comply with scheduled visits, laboratory tests, and other trial procedures; (8) ability to understand the provided information and provide written informed consent; (9) affiliation to a social security system; and (10) life expectancy of at least 12 months.

#### Exclusion Criteria

The exclusion criteria are age of <60 years and a diagnosis of severe psychiatric or neurological disorders that might impair the ability to provide informed consent.

### Study Time Points and Procedures

After the patient has signed the written informed consent form, she will follow the study procedures set for each time point, as shown in Table 2. Table 3 shows all self-administered scales that the patient has to complete for each study time point. These procedures are the same for both the standard practice and enhanced monitoring groups.

**Table 2:** Summary of scheduled procedures.

Procedure	Day −15: T0 (screening)^a^	T1 (baseline)^a^	Day 0: treatment start	Month 3: T2	Month 6: T3	Month 9: T4	Month 12: T5	14-25 days after the end of treatment^b^
ICF^c^ signature	✓							
Diagnosis	✓							
Personal and family medical history (including diagnosis of severe psychiatric disorders)	✓							
Smoking habits		✓						✓
Comorbidities	✓							
Oncological visit		✓			✓		✓	
Psychological visit^d^ (if the center has a psychological support service)		✓		✓	✓	✓^e^	✓	
Cardiological visit (if needed)	✓	✓		✓	✓	✓^e^	✓	
BP^f^ and HR^g^ measurement		✓		✓	✓	✓^e^	✓	
Routine blood analysis (ie, hematology and biochemistry)^h^		✓		✓	✓	✓	✓	
Echocardiographic assessment^i^		✓		✓	✓	✓^e^	✓	
ECG^j^ assessment		✓		✓	✓	✓^e^	✓	
Plasma troponin I level assessment	✓^k^	✓^k^		✓	✓	✓	✓	
Mammography (if the patient did not undergo mastectomy)		✓					✓	
Plasma BNP^l^ assessment		✓		✓	✓	✓	✓	
Plasma myeloperoxidase and high-sensitivity CRP^m^ assessment (if available)		✓		✓	✓	✓	✓	
Inclusion and exclusion criteria checklist	✓							
Blood sample for genetic analysis		✓						
Plasma sample for micro-RNA analysis		✓						✓
Stool sample		✓						✓
Oncological treatment (plan and changes) and cycle			✓	✓	✓	✓	✓	✓
Information on treatment status (ongoing or completed)				✓	✓	✓	✓	
Concomitant medications	✓	✓	✓	✓	✓	✓^e^	✓	✓
GSRS^n^		✓						✓
Patient lifestyle questionnaire		✓						✓
Verification of completion of self-administered scales and questionnaires (ePsycHeart section)^o^		✓	✓	✓	✓	✓	✓	
Cognitive effect assessment (if the center has specialized personnel)		✓			✓		✓	
Verification of collection of data from wearable devices				✓	✓			
Collection of information on patient’s out-of-pocket expenses					✓		✓	
Hand grip test		✓			✓		✓	
AE^p^ assessment		✓	✓	✓	✓	✓	✓	✓

^a^May be 1 or 2 visits.

^b^At the first visit after the end of each treatment, which should be within 14 to 25 days. The time of these sample collections will vary in relation to the therapeutic schedule of the patient.

^c^ICF: informed consent form.

^d^These visits can be conducted in person or remotely if the center can involve the required experts. The psychological visit consists of meeting the patient in person (if already at the clinical center) or remotely to touch base about the study—how they feel about it and whether they would like something to be different or modified. During the visit, a motivational intervention will also be carried out to minimize dropouts as much as possible.

^e^Only if possible.

^f^BP: blood pressure.

^g^HR: heart rate.

^h^Including cholesterols, glucose levels, and renal and liver function tests and interleukin-6, tumor necrosis factor, HR variability, C-reactive protein, fibrinogen, and ferritin if available.

^i^Including right and left ventricle dimensions and functions and diastolic function.

^j^ECG: electrocardiogram.

^k^Troponin can be analyzed either at screening or at baseline.

^l^BNP: brain natriuretic peptide.

^m^CRP: C-reactive protein.

^n^GSRS: Gastrointestinal Symptom Rating Scale.

^o^Table 3 provides a precise list of the questionnaires and scales administered at each time point.

^p^AE: adverse event.

**Table 3:** Self-administered scales for each study time point.

Psychological or behavioral assessment	Day −15: T0^a^	T1 (baseline)^a^	Day 0: treatment start	Month 3: T2	Month 6: T3	Month 9: T4	Month 12: T5
Quality of life (EORTC^b^ QLQ-30^c^ and QLQ-BR23^d^)		✓		✓	✓	✓	✓
Fatigue (FACIT^e^-Fatigue)		✓		✓	✓	✓	✓
Resilience (Brief Resilience Scale)		✓		✓			✓
Anxiety and depression (PHQ-4^f^)		✓		✓	✓	✓	✓
Emotion regulation questionnaire (ERQ^g^)		✓		✓			✓
LOT-R^h^		✓		✓			✓
Life satisfaction (single-item life satisfaction measure)		✓	✓	✓	✓	✓	✓
Perceived social support (MSPSS^i^)		✓		✓			✓
Self-control and self-management (SCMS^j^)		✓		✓			✓
Coping strategies (CBI-B^k^)		✓		✓	✓	✓	✓
Post Traumatc Stress disorder (IES-R^l^)		✓		✓			✓
Perceived Stress (PSS^m^)		✓		✓	✓	✓	✓
Positive and negative affect (B-PANAS^n^)		✓		✓			✓
Burden for family caregivers (BSFC-s^o^)—optional		✓		✓	✓	✓	✓
Nutritional questionnaire		✓		✓			✓

^a^May be 1 or 2 visits.

^b^EORTC: European Organisation for Research and Treatment of Cancer.

^c^QLQ-30: Quality of Life Questionnaire.

^d^QLQ-BR23: Quality of Life Questionnaire–Breast Cancer.

^e^FACIT: Functional Assessment of Chronic Illness Therapy.

^f^PHQ-4: 4-item Patient Health Questionnaire.

^g^ERQ: Emotion Regulation Questionnaire.

^h^LOT-R: Life Orientation Test–Revised.

^i^MSPSS: Multidimensional Scale of Perceived Social Support.

^j^SCMS: Self-Control and Self-Management Scale.

^k^CBI-B: Cancer Behavior Inventory–Brief Version.

^l^IES-R: Impact of Event Scale–Revised.

^m^PSS: Perceived Stress Scale.

^n^B-PANAS: Brief Positive and Negative Affect Schedule,

^o^BSFC-s: short version of the Burden Scale for Family Caregivers.

### Instruments and Measures

All recruited patients will receive additional supportive care in conjunction with standard care. Patients in the standard practice group and those in the enhanced behavioral and psychological monitoring group of the study will receive wearable devices and complete an initial evaluation on the CARDIOCARE mobile app.

#### The CARDIOCARE Mobile App

The CARDIOCARE mobile app is installed on the Motorola smartphone provided to patients, which they will have to return after use (end of month 6—T3). The CARDIOCARE app allows access to 2 different settings containing specific modules (described in this section) depending on the group—standard practice or enhanced behavioral and psychological monitoring—the patient is assigned to.

The Cognitive Stimulation, Education and Training, Vision and Hearing, and Psychology and Well-being (except for the Questionnaires submodule) modules will only be accessed by the intervention arm of the study.

The six modules are (1) Mobility and Vitality, (2) Cognitive Stimulation, (3) Education and Training, (4) Geriatric Syndrome Assessment, (5) Vision and Hearing, and (6) Psychology and Well-being.

The Mobility and Vitality module contains 4 submodules, which are the nutrition questionnaire, physical activity, the cardio recording session, and the hand grip session. Together, these submodules will provide a collection of graphs—such as bar and pie charts—representing data on the patient’s overall functional well-being, sleep duration, physical activities, and average HR, as well as proxies of the patient’s stress levels.

The Cognitive Stimulation module includes 4 cognitive games, which are Find the Word, tic tac toe, Color Beans, and Drawing. If played consistently, such games can foster the patient’s attention, concentration, and working memory capacities, thereby counteracting the cognitive impairment often found in patients undergoing oncological treatments [21].

The Education and Training module includes 2 submodules. The first one is Breast Cancer Material, which contains patient-oriented information related to breast cancer and cardiotoxicity and on the potential psychological and social implications that breast cancer and its treatment can have. This submodule provides a deeper awareness about the clinical implications of the disease and guidance for the patients to preserve their psychological well-being. The second submodule is named Training and contains a video tutorial for patients to improve their balance and reinforce their muscle strength.

The Geriatric Syndrome Assessment module includes 2 submodules. The first one is Incontinence Management, which will allow each patient to report urination incidents, perform guided exercises for pelvic floor strengthening, and set reminders for toilet use. The second one is the Fall Mitigation submodule, where the patient can access the Otago program, a set of exercises designed to help them enhance and restore their balance [22].

The Vision and Hearing module includes the Vision Test and Hearing Test submodules. The first is an assessment of the patient’s vision capacity that was designed based on the Snellen test guidelines. The second one is an assessment of the patient’s hearing capability designed based on the whisper test guidelines.

Finally, the Psychology and Well-being module includes behavioral and psychological interventions corresponding to 5 submodules: expressive writing, BPS, ABCDE (A=activating event or situation; B=beliefs; C=consequences; D=disputation of beliefs; E=effective new approach), Biofeedback Session, and Questionnaires. The interventions in the first 4 modules aim to mitigate the psychological risk factors associated with cardiotoxicity development and deterioration of the patient’s QoL. The fifth submodule, Questionnaires, includes 15 self-reported measures that will provide critical information on the patient’s levels of perceived stress, resilience, anxiety and depression, self-management and emotion regulation capacity, coping skills, perceived social support, satisfaction with life, QoL, and levels of optimism (see Table 3 for the list of questionnaires used).

#### Wearable Devices

The CARDIOCARE mobile app, described previously, will process data derived from 2 technologically advanced wearable devices: the Garmin Venu SQ watch and the Polar H10 sensor. The Garmin Venu SQ watch will collect data on the patient’s HR (resting and high), body battery (percentage and charged or drained), stress (rest, low, medium, and high), intensity minutes (per week, for the current day, and goal), steps (for the current day and goal), calories (total for the current day, resting, and active), and respiration (breaths per minute, sleep average, and waking average). The Polar H10 sensor is a chest strap measuring highly detailed and accurate information about the patient’s HR and electrocardiogram.

The patient is required to wear the Garmin Venu SQ watch for the first 6 months of the study and to wear the Polar H10 sensor for approximately 30 minutes every 2 weeks. At the end of month 6 of the study (T3), each patient must return the 2 wearable devices and the smartphone to their clinical center.

Patients will also be asked to use the hand grip dynamometer at T1 and T3 to assess their hand grip strength during their cardiological or oncological visit.

### Statistical Procedure

#### Overview

The CARDIOCARE study proposes a comprehensive approach to measure the efficacy of the eHealth intervention by assessing its impact on 2 primary outcomes: intrinsic capacity and QoL in older patients with breast cancer. A prospective, multicentric study with 750 patients aged ≥60 years assigned to standard practice or enhanced behavioral and psychological monitoring groups will be conducted.

The enhanced behavioral and psychological monitoring group receives access to the eHealth psychological, behavioral, and functional modules on the CARDIOCARE mobile app. The primary measurements are intrinsic capacity, evaluated using physiological, physical, and cognitive parameters through wearable devices (Garmin Venu SQ watch and Polar H10 sensor) for the first 6 months, and QoL, measured through self-reported questionnaires integrated into the CARDIOCARE app covering aspects such as stress, resilience, and emotional regulation. Multimodal data, including clinical (cardiac imaging and biochemical markers), psychological (anxiety and depression scales), and behavioral data are analyzed to obtain a holistic understanding of the patient. The data are collected at baseline (T0) and every 3 months up to 12 months (T5).

This longitudinal approach captures changes over time. The intervention group undergoes targeted psychological, behavioral, and functional eHealth interventions. The comparison between the intervention and control groups will provide useful information on the interventions’ impact on mitigating cardiotoxicity and improving QoL.

As of October 17, 2024, a total of 375 patients have been enrolled in the clinical study. For all these patients, data of different categories are collected and used in the training of the risk stratification model of cardiotoxicity and the prediction model of QoL. Once the model validation phase is complete, the results will be made available and presented in a subsequent publication.

#### Statistical Considerations Regarding the Design

The primary statistical analysis will use an intention-to-treat approach. Therefore, all patients involved in the study will be included in the final analysis. Results will be presented using descriptive statistics (mean, SD, median, and range for continuous variables and proportions for nominal variables). Patient baseline characteristics will be compared using a 2-sample *t* test or a nonparametric test for continuous variables, and the Pearson chi-square test will be used for qualitative variables. All statistical analyses will be conducted using 2-tailed tests and adopting a 5% significance level. The effect of the interventions on the primary end point will be analyzed using the Cox proportional hazards model. Point estimates and 95% CIs will be calculated for the hazard ratio of the standard practice group versus the enhanced behavioral and psychological monitoring group. Any statistically significant difference between the 2 groups at baseline will be balanced by the multivariable adjustment. A logistic regression or Cox proportional hazards model will also be used to evaluate the effect of the interventions on the secondary end points in the 2 groups. Continuous variables such as those obtained from imaging and biochemical and molecular biomarkers will be summarized descriptively at baseline and follow-up visits. The differences between the 2 groups during the different follow-up visits will be evaluated using the analysis of covariance considering the corresponding baseline measurement as a covariant.

Finally, for the cost-effectiveness analysis, the health and QoL outcomes observed during the study will be used to calculate the QALYs. These QALYs will be combined with cost data in a cost-utility analysis to estimate the cost per QALYs associated with the implementation of the CARDIOCARE model and the eHealtHeart interventions. The incremental cost-effectiveness ratio will also be calculated to evaluate the CARDIOCARE model’s effectiveness compared to current care practices. Therefore, the incremental cost-effectiveness ratio, which will be calculated using a threshold approach, will allow us to better understand how to distribute and allocate the different health care resources.

#### Sample Size Considerations

To identify the adequate sample size for the CARDIOCARE prospective study, a power analysis was conducted with 2-tailed α=.05 and 1 – β=.80. On the basis of this, and considering the 21% incidence of cardiotoxicity in our target population and the expected 10% dropout rate, it was estimated that a total of 736 patients (ie, 368 patients in the intervention arm and 368 patients in the control arm) would be large enough to detect a 40% relative risk reduction in the incidence of increased levels of troponin I (taken as a cardiotoxicity proxy) in this population. Such a sample size would also allow for the evaluation of the effectiveness of the behavioral and psychological interventions integrated into the study in mitigating, preventing, or delaying the onset of cardiotoxicity associated with oncological therapies for older patients with breast cancer.

The sample size calculation was followed by the Fisher exact test, conducted using the G*Power software (version 3.1.9.4). Considering our power analysis results and the number of patients affected by breast cancer each year, all clinical centers in the CARDIOCARE consortium agreed on the final recruitment goal of 750 patients (375 for the intervention arm and 375 for the control arm).

### Data Collection, Storage, and Security

A well-defined data management plan has been submitted, providing detailed information on the procedures that will be implemented for data collection, storage, protection, retention, reuse, or destruction, complying with the General Data Protection Regulation.

By design, a robust data protection and security strategy will be implemented in all data collection and storage procedures in the CARDIOCARE platform. As part of the CARDIOCARE data management and high-performance computing, an eCRF—accessible by all clinical partners—has been developed to serve as the data entry tool for all patient data. eCRF data will be uploaded to and stored encrypted on the platform and systematically backed up in external redundant array of inexpensive disk drives. An audit log operation will be implemented to view the users’ access history to the system, enabling the detection of any potential data security breaches. After final quality checks at the end of the prospective study, the eCRF will be frozen and exported to the technical partners for statistical analysis performance.

All members of the CARDIOCARE consortium will take appropriate organizational and technical measures to prevent any event of abuse, accidental loss, destruction, or damage of collected data, enabling the reinstatement of the system promptly if necessary.

Data quality, completeness, and integrity will be ensured through visits from the Independent Data Monitoring Committee members, who will certify, in each clinical center, the files’ consistency, adherence to the study protocol and good clinical practice guidelines, the accuracy of the eCRF forms, and the compliance with safety reporting. Investigators of each clinical center will facilitate the Independent Data Monitoring Committee members’ jobs by cooperating and enabling direct access to all data sources to be verified.

Strict confidentiality of all personal and study-related data is ensured. All recruiting centers will sign an agreement document detailing their commitment toward complying with the relevant laws, regulations, codes of practice, and obligations to publication.

### Ethical Considerations

All involved clinical centers submitted the new version (1.2) of the CARDIOCARE prospective study protocol to their ethics committees and obtained official approval as follows: BOCOC on January 25, 2024 (approval EEBK/ETT/2022/58); KSBC on October 12, 2023 (approval 2023-0026201-413152); IEO on November 22, 2023 (approval R1754/22-IEO 1874); IOL on December 12, 2023 (approval ERIDEK-0038-2023); NKUA on November 24, 2023, for Aretaieion Hospital (approval 456/14-10-2022) and December 5, 2023, for Attikon Hospital (approval 683/22-11-2022); and UOI on November 23, 2023 (approval EBA683/22-11-2022).

This multicentric prospective study has been designed to comply with national (ie, good clinical practice) and international (ie, the Declaration of Helsinki) regulations of proper ethical research involving human participants with written informed consent, which is obtained from all participating patients. Participants are clearly informed about the possibility to opt out of the study at any time. The collected data are anonymized and deidentified, ensuring the protection of privacy and confidentiality. No compensation is provided for participation in the study.

In particular, the study conduct is in line with the following regulations and norms: the Declaration of Helsinki ethical principles for medical research involving human participants, revised in October 2013; the Convention for the Protection of Human Rights and Dignity of the Human Being With Regard to the Application of Biology and Medicine: Convention on Human Rights and Biomedicine (Oviedo, 1997); the *International Ethical Guidelines for Biomedical Research Involving Human Subjects* by the Council for International Organizations of Medical Sciences in collaboration with the World Health Organization, revised in 2016; and the Belmont Report: Ethical Principles and Guidelines for the Protection of Human Subjects of Biomedical and Behavioral Research (US Department of Health, Education, and Welfare publication [DHEW-05-78-0012], Washington, District of Columbia, 1978).

## Results

The active phase of the recruitment process began in May 2023, and preliminary results will be published in a scientific journal and made available for consultation on the CARDIOCARE project website. Data analysis and dissemination of the study results will take place in 2025.

Patient recruitment started in May 2023, and by October 17, 2024, a total of 50% (375/750) of the target number of patients had been recruited (Table 4).

**Table 4:** Number of recruited patients in total and per clinical center.

Clinical center	Current number of patients in the study	Total target	Progress, %	Number of dropouts	Dropouts at each clinical center, %
BOCOC^a^	110	120	91.7	14	12.7
NKUA^b^	109	195	55.9	14	12.8
UOI^c^	42	60	70	6	14.3
IEO^d^	73	125	58.4	4	5.5
KSBC^e^	23	125	18.4	1	4.3
IOL^f^	19	125	15.2	4	21.1
Total	375	750	50	43	11.5

^a^BOCOC: Bank of Cyprus Oncology Centre.

^b^NKUA: National and Kapodistrian University of Athens.

^c^UOI: University of Ioannina.

^d^IEO: European Institute of Oncology.

^e^KSBC: Karolinska University Hospital.

^f^IOL: Institute of Oncology Ljubljana.

In October 2023, a new amended version of the protocol (version 1.2) was approved. In the face of emerging new evidence [23,24], an amendment was proposed regarding the definition of cardiotoxicity and inclusion criteria related to age and treatment. More specifically, the identification of cardiotoxicity also includes so-called MACEs, and the intrapatient assessment of MACEs, taxanes, and endocrine therapies with or without cyclin-dependent kinase 4 or 6 inhibitors were included among the inclusion criteria. In addition, the inclusion criterion of age decreased from ≥65 to ≥60 years.

## Discussion

### Expected Findings

The CARDIOCARE study is expected to demonstrate the feasibility and utility of a risk prediction model tailored to assess the likelihood of cardiotoxicity in older patients with breast cancer. It is hypothesized that this model, integrated with the CARDIOCARE mobile app and wearable technologies, will not only identify individuals at high risk more accurately but also provide actionable, personalized, and tailor-made interventions that help manage and mitigate risks associated with cardiotoxicity. In addition, the findings are anticipated to highlight the model’s role in enhancing intrinsic capacity, QoL, and cost-effectiveness for both patients and health care systems.

The CARDIOCARE model is expected to bridge critical gaps in the early identification and management of cardiotoxicity, especially in older patients with breast cancer, a group at significant risk due to age-related factors, polypharmacy, and oncological treatments. The mobile app is designed to offer continuous, home-based care by promptly alerting patients and clinicians when health parameters are suboptimal. This facilitates timely interventions that can preserve intrinsic capacity, improve physical and psychological well-being, and enhance overall QoL. This study is also anticipated to underscore the potential for digital health tools to provide a more integrated and comprehensive picture of patient health compared to traditional electronic health records.

The CARDIOCARE risk prediction model for cardiac toxicity will allow health care providers to identify and monitor trajectories across the field of cardiotoxicity and, thus, advise and intervene promptly on patients at high risk while saving resources on those presenting a low risk of developing cardiotoxicity. The model will be particularly tailored to detect the risk of cardiotoxicity in older patients with breast cancer, whose risk of cardiovascular disease is almost the same as that of breast cancer relapse [23,25]. Furthermore, even when the cancer is not fatal, older women with breast cancer are especially prone to develop cardiac morbidity as a collateral effect of oncological treatments [26]. Indeed, in this clinical population, cancer treatment cardiotoxic effects are often added to baseline age-related factors and preexisting morbidity, leading to drug interaction complications resulting from polypharmacy [27,28], poorer health outcomes, and intrinsic capacity and QoL deterioration [29].

Considering the aforementioned factors, the CARDIOCARE mobile app, with its contents and health monitoring system, will provide older women with breast cancer with the opportunity to learn how to best manage their multimorbidity while preserving and enhancing their physical and psychological intrinsic capacity as well as their overall QoL, including their relationship with others. They will also benefit from the continuous and home-based care that the mobile app provides by notifying them promptly when one or more health parameters are suboptimal, and clinician-developed suggestions on how to intervene to improve such parameters will follow.

In addition, this study provides an opportunity to test the feasibility of digital tools (devices and mobile app) among this specific population of older patients (aged ≥60 years) and identify the possible areas that need improvement so that, in the future, these types of digital tools and interventions can be successfully applied to the specific segment of patients considered in this study.

Health care providers will also vastly benefit from the health monitoring and technologically advanced tools used in this prospective study. Such tools, including the mobile app, will provide clinicians with more integrated information on their patients’ health than the information they can obtain through standard electronic health records. The CARDIOCARE mobile app and the wearable devices used in this study will, indeed, inform health care providers about several characteristics of their patients (psychological, behavioral, cognitive, and functional) that, based on the most updated scientific literature, are particularly relevant to determine the risk of older patients with breast cancer of developing cardiac toxicity when exposed to oncological therapy [6,30-32]. Furthermore, by being informed on their patients’ intrinsic capacity (physical and psychological) and other QoL-related aspects, health care providers can more easily and quickly identify each patient’s care gaps and specific needs and develop new and more integrated best practices, facilitating the implementation of personalized, patient-centered care.

The innovative CARDIOCARE model of care and its risk prediction model for cardiac toxicity hold promise from a socioeconomic perspective as well. It is to be underlined that almost 40% of public spending in the health care sector concerns people aged >60 years, with long-term care and hospital admissions being the primary cost drivers [33]. As health care systems that successfully provide effective community-based care and services are likely to significantly optimize their public spending [33], health care institutions and insurance companies are seeking new ways to decrease treatment costs for chronic diseases such as cancer.

Moreover, previous research has highlighted considerably higher mortality rates among patients with breast cancer of a low socioeconomic status (ie, education, employment, and income) compared to those whose socioeconomic status is higher [34,35]. This is a critical datum as socioeconomic disadvantage may result in later-stage diagnosis and poorer access to and quality of care. Using technologically advanced tools—provided to patients by the clinical centers—able to consistently monitor several health parameters beyond the time the patient stays at the clinical center, the CARDIOCARE health care model attempts to address the economic and societal challenges that afflict both our health care system finances and patients of a lower socioeconomic status. In addition, once the prospective study is concluded and the data are analyzed, a cost-effectiveness analysis will be conducted comparing the CARDIOCARE health care model to current practices.

### Potential Limitations

This study’s psychological and behavioral monitoring is based on the use of a mobile app and wearable sensors, which may be problematic for some older adult users who are not familiar with or proficient in the use of this type of technology. Assistance and training in the use of the technology are provided at the recruitment stage, but the continued engagement with the app over the study period and compliance with the use requirements could pose significant obstacles to data collection. To circumvent this problem, the implementation of the study protocol includes frequent interaction between clinical staff and participants, as well as encouraging the involvement of family members to assist in maintaining compliance with the study protocols. This may result in a bias introduced by the level of technological dexterity of the patient, support in this regard from family members, or willingness to initiate communication with and seek help from clinical staff.

In addition, as with all multicenter studies, the study protocol is strictly applied and monitored at each clinical center to maintain consistency between centers. All equipment (eg, mobile phones and wearables) is the same and is provided through the CARDIOCARE project. Assessments of cardiotoxicity and subclinical cardiotoxicity are performed by a consensus group of cardiologists from each clinical center using all the available blood tests, echocardiograms, and treatment and patient history data. However, differences between populations, cultures, and environmental factors will be considered in the analysis plan to minimize any inherent bias.

Finally, a potential limitation of this project concerns the fact that the study follow-up period is limited to 12 months from the enrollment date to encompass both acute and early (within the first year) cardiotoxicity, defined based on imaging and biological biomarkers. Late cardiotoxicity (occurring up to several years after treatment) is not considered in this study.

### Conclusions

The CARDIOCARE prospective study will contribute to developing new best practice guidelines for managing older patients with breast cancer and multimorbidity while preserving their intrinsic capacity and improving their QoL. Furthermore, the CARDIOCARE mobile app and the wearable devices will enable clinicians to identify trajectories across the cardiotoxicity disease continuum, allowing for preventative interventions for patients at higher risk. This innovative health care approach will also benefit the health care system, which allocates nearly 40% of its resources to patients aged >60 years, with long-term care and hospital admissions being the primary cost drivers.

## Abbreviations

BOCOC: Bank of Cyprus Oncology Centre
BPS: best possible self
DICOM: Digital Imaging and Communications in Medicine
eCRF: electronic case report form
HER2: human epidermal growth factor receptor 2
HR: heart rate
IEO: European Institute of Oncology
IOL: Institute of Oncology Ljubljana
KSBC: Karolinska University Hospital
LVEF: left ventricular ejection fraction
MACE: major adverse cardiovascular event
NKUA: National and Kapodistrian University of Athens
QALY: quality-adjusted life year
QoL: quality of life
UOI: University of Ioannina
